# Cytochrome P450 2C19*2 polymorphism in patients with stable coronary heart disease and risk for secondary cardiovascular disease events: results of a long-term follow-up study in routine clinical care

**DOI:** 10.1186/1471-2261-13-61

**Published:** 2013-08-27

**Authors:** Dietrich Rothenbacher, Michael M Hoffmann, Lutz P Breitling, Iris Rajman, Wolfgang Koenig, Hermann Brenner

**Affiliations:** 1 Institute of Epidemiology and Medical Biometry, Ulm University, Helmholtzstr 22, Ulm D-89081, Germany; 2 Division of Clinical Epidemiology & Aging Research, German Cancer Research Center, Heidelberg, Germany; 3 Department of Clinical Chemistry, University Medical Center Freiburg, Freiburg, Germany; 4 Novartis Institute of Biomedical Research, Basel, Switzerland; 5 Department of Internal Medicine II-Cardiology, University of Ulm Medical Center, Ulm, Germany

## Abstract

**Background:**

CYP2C19*2 polymorphism is related to metabolizer phenotypes resulting in reduced effectiveness in converting the antiplatelet drug clopidogrel to active drug. An association of the genotype itself with adverse outcomes is discussed. We investigated the prognostic value of carriage of the CYP2C19*2 allele in a high risk group of patients with prevalent coronary heart disease (CHD) at baseline during long-term follow-up under conditions of routine clinical care.

**Methods:**

In n=1050 patients with stable CHD at baseline genotyping of CYP2C19 allele *2 (rs4244285; 681G>A) was performed. The Cox-proportional hazards model was employed to investigate the association of CYPC19*2 allele status with cardiovascular disease (CVD) events during eight year follow-up. The analysis was also performed in patients who did not take clopidogrel or ticlopidin.

**Results:**

Only the very few patients homozygous for a loss-of-function variant of CYP2C19, allele *2 (2.6%), had a statistically significantly higher incidence rate for secondary CVD events during long-term follow-up than wild-type carriers (50.8 versus 21.5 per 1000 patients years; rate for heterozygous carries 17.2 per 1000 patient years). The hazard ratio after adjustment for covariates compared to the wild-type carriers was 2.59 (95% confidence interval (CI) 1.27-5.28) and 0.80 (95% CI 0.52-1.23) for homozygous and heterozygous allele carriers, respectively.

**Conclusions:**

In this medium-size group of patients with stable CHD homozygous carriers of the loss-of-function allele CYP2C19*2 were at increased risk for subsequent CVD events during 8 year follow-up independent of other risk factors. As only few patients carried the homozygous loss-of-function variant and we found overall no evidence for improved clinical utility, a benefit of genotyping in this patient population seems unlikely.

## Background

In the meantime a one-year dual antiplatelet therapy is standard treatment for patients with acute coronary syndrome (ACS) and percutaneous coronary intervention (PCI) for secondary prevention of thrombo-occlusive cardiovascular complications [1]. Various genetic factors have been identified that seem to hamper this preventive concept, especially polymorphisms related to metabolizer phenotype [2]. Cytochrome P450 2 C19 is a mixed-function oxidase of the P450 cytochrome family that is mostly involved in metabolizing xenobiotics in the body, and in synthesis of cholesterol and other lipids. There are several polymorphisms for this gene which influence the presence and activity of the enzyme.

The most common alleles that result in the poor metabolizer phenotype are the *2 and *3 alleles. The carriage of *2 allele has been described in 95% of poor metabolizers [3]. Poor metabolizers have an impaired effectiveness in converting the antiplatelet drug clopidogrel to the active drug to exert its antiplatelet effect. In a cohort of healthy subjects carriage of at least one CYP2C19*2 reduced-function allele resulted in a relative reduction of 32.4% in plasma exposure to active clopidogrel [4]. In a separate cohort of patients with ACS *2 carriers also had a higher rate of major cardiovascular events compared to non-carriers [4]. In contrast, Pare and colleagues presented data from two large randomized controlled trials showing no association between increased cardiovascular risk and CYP2C19 loss of function variants [5]. The latter may have been caused by the comparison of loss-of-function allele carriers taking clopidogrel with those taking placebo, which showed that carriers taking clopidogrel still had improved outcomes compared to placebo. A collaborative meta-analysis conducted by Mega et al. [6] including patients with acute coronary heart disease (CHD) and treated with clopidogrel showed that carriage of even one reduced-function CYP2C19 allele was associated with a significantly increased risk of adverse cardiovascular events.

Current evidence suggests that the CYP2C19 polymorphisms may be clinically relevant mainly through the effects on metabolism of clopidogrel prodrug, but further data are needed to determine if other mechanisms are possible too and whether routine testing at the point of care may be beneficial for the patients [7]. The Food and Drug Administration (FDA) released a Boxed Warning in the year 2010 that patients with a poor metabolizer phenotype may not effectively metabolize clopidogrel prodrug and therefore may not experience its full benefits [8]. Although the impact of the CYP2C19 polymorphisms on the functional consequences on enzyme activity are well understood [2,3] and an increased risk for major adverse cardiovascular risk in subjects taking clopidogrel has been suggested [4], the question whether variants of CYP2C19 are associated with an adverse outcome itself, independent of clopidogrel intake is still discussed. This is relevant given that antiplatelet therapy is recommended only for one year for patients with ACS or PCI [1]. Therefore, more information is needed to further understand the relationship between genotype, clopidogrel intake and subsequent risk for secondary cardiovascular disease events, especially in high CHD risk-patients from routine clinical care.

We investigated the prognostic value of carriage of the CYP2C19*2 allele in a high risk group of patients with prevalent CHD at baseline during long-term follow-up of eight years under conditions of routine clinical care and considering other concurrent risk factors. We were also investigating whether carriage of the CYP2C19*2 allele itself is associated with an adverse outcome in patients not taking dual-platelet therapy.

## Methods

### Study population

All patients with CHD (International Classification of Diseases, 9^th^ Rev. codes. 410–414) aged 30–70 years and participating in an in-hospital rehabilitation program between January 1999 and May 2000 in two cooperating hospitals (Schwabenland-Klinik, Isny and Klinik im Südpark, Bad Nauheim, Germany) were enrolled in the study (response at baseline 58%). In Germany, all patients after an acute coronary syndrome (ACS) or elective coronary artery bypass grafting (CABG) are offered a comprehensive in-hospital rehabilitation program following their discharge from the acute care hospital. The aim of this 3-weeks program is the reduction of cardiovascular risk factors, improvement of health related quality of life, and the preservation of the ability to work (the latter only if a subject was still at work at the onset of disease, otherwise the prevention of need for nursing care). This in-hospital rehabilitation program usually starts approximately three weeks after the acute event or CABG. In the current study, only patients who were admitted within three months after the acute event or CABG were included. Further details can be found elsewhere [9].

All subjects gave written informed consent. The study was approved by the Ethics Boards of the Universities of Ulm and Heidelberg and of the Physicians’ chamber of the States of Baden-Wuerttemberg and Hessen (Germany).

#### Data collection

At the beginning of the in-hospital rehabilitation program all subjects filled out a standardized questionnaire containing socio-demographic information and medical history. In addition, information was taken from the patients’ hospital charts. In all patients active follow-up was conducted 1, 3, 4.5, 6 and 8 years after discharge from the rehabilitation centre. Information was obtained from the patients using a mailed standardized questionnaire. Information regarding secondary cardiovascular disease (CVD) events and treatment since discharge from the in-hospital rehabilitation clinic was obtained from the primary care physicians by means of a standardized questionnaire. If a patient had died during follow-up, the death certificate was obtained from the local Public Health Department and the main cause of death was coded according to the International Classification of Diseases (ICD-9 pos. 390–459: ICD-10 pos. I0-I99 and R57.0). Secondary CVD events were defined as CVD as the main cause of death (as stated in the death certificate), non-fatal myocardial infarction (MI), or non-fatal ischemic stroke. All non-fatal secondary events were reported by the primary care physicians.

#### Laboratory methods

Genotyping of one single-nucleotide polymorphism (SNP) defining the CYP2C19*2 allele (rs4244285;681G>A) was performed with a TaqMan assay of ABI (C__25986767_70; Applied Biosystems, Foster City, CA) from stored peripheral blood DNA. The deviation from Hardy-Weinberg equilibrium was non-significant.

Blood at baseline was drawn at discharge from the rehabilitation centre on average 43 days after the acute event (1st quartile 36 days, 3rd quartile 51 days) in a fasting state under standardized conditions and stored at −80°C until analysis. C-reactive protein (CRP) concentrations were measured by immunonephelometry on a Behring Nephelometer II (N Latex CRP mono Dade-Behring, Marburg). Cystatin C was determined on the same device (Dade-Behring, Marburg). NT-proBNP was measured by electrochemiluminescence on an Elecsys 170 (Roche Diagnostics, Mannheim, Germany). Inter-assay coefficients of variation (CVs) were 4.1% for CRP, 3.8% for cystatin C, between 3% and 7% for NT-proBNP. All markers were measured in a blinded fashion. Creatinine, blood lipids and leukocyte count were done by routine methods in both participating hospitals.

#### Statistical methods

The study population was described with respect to various sociodemographic and medical characteristics. If variables were normally distributed, means and standard deviation were given, otherwise median and interquartile range. We also calculated a modified version of the GRACE score as published by Eagle and colleagues to describe the risk for subsequent mortality [10]; the only variable among the nine variables contained in the score we could not include was ST-segment depression at the index events, since we did not have information on all subjects for it. The associations of sociodemographic characteristics, various cardiovascular risk factors, severity of cardiovascular disease, and medication with CYP2C19*2 allele status (GG, AG, AA; AA or AG versus GG) were quantified by means of the nonparametric Kruskal-Wallis test (an exact test was used if cell number was n< 5). In addition mean values, adjusted for age and gender, were calculated for categories of CYPC19*2 allele status and body mass index, blood lipids, CRP, IL-6, creatinine clearance, cystatin C, NT-proBNP and quantified by a respective p-value.

The relation of CYP2C19*2 allele status with CVD events during follow-up was assessed by the Kaplan-Meier and life table method and quantified by means of the log-rank test. Then the Cox proportional hazards model was employed to assess the independent association of CYP2C19*2 allele status with the risk of secondary CVD events (hazard ratios, HR, and their 95% confidence intervals, CI). A basic model was adjusted for age (years) and gender. In a second step, beside the main factor CYP2C19*2 allele status, the variables age and gender, the following potential confounders were considered in multivariable analyses: hospital site, body mass index (BMI, kg/m^2^), school duration (< 10 years, ≥10 years), smoking status (never, current, ex-smoker), history of diabetes mellitus (yes, no), initial management of CHD (conservative, percutaneous coronary intervention, PCI, CABG), left ventricular function (degree of impairment: no or little, modest severe, unknown), intake of lipid-lowering drugs (yes, no), HDL-cholesterol (mg/dl), LDL-cholesterol (mg/dl), and NT-proBNP, and finally intake of clopidogrel. Finally we excluded all patients with a clopidogrel and ticlopidine intake at baseline and one-year follow-up to investigate the association of the CYP2C19*2 allele itself with the outcome. All statistical procedures were carried out with the SAS statistical software package (release 8.2, Cary, NC (USA), SAS Institute Inc. 1999).

## Results

Overall, 1,206 patients with a diagnosis of CHD within the past 3 months were included at baseline during the in-hospital rehabilitation program. Eight-year follow-up information was complete for 1,050 patients (87.1%, all of Caucasian origin). The main characteristics of the study population are shown in Table 1. The mean age of the study population at baseline was 58.8 years, most of the patients were male (85.0%). A history of myocardial infarction or diabetes was reported by 58.2% and 17.7%, respectively. A 3-vessel disease was present in 42.8% of the CHD patients at baseline. For the CYP2C19*2 polymorphism 26.6% were heterozygous, and 2.6% were homozygous poor metabolizers with a loss-of-function gene.

**Table 1:** Sociodemographic, clinical, and laboratory characteristics in 1,050 patients with clinically manifest coronary heart disease

**Characteristics at baseline**	
Age (years) (μ, SD)	58.8 ± 8.0
Men, n (%)	892 (85.0%)
CYP2C19C*2 allele frequency	
- AA	27 (2.6%)
- AG	279 (26.6%)
- GG	744 (70.9%)
History of myocardial infarction, n (%)	611 (58.2%)
History of heart failure, n (%)	128 (12.6%)
Clinical score (angiographic evaluation)	
- 1 vessel disease	255 (24.3%)
- 2 vessel disease	279 (26.6%)
- 3 vessel disease	449 (42.8%)
- unknown	52 (5.0%)
School education <10 yrs., n (%)	625 (59.5%)
Body mass index (kg/m^2^), (μ, SD)	27.1 ± 3.5
History of diabetes, n (%)	184 (17.7%)
Total cholesterol (mg/dl) (μ, SD)	168.9 (32.6)
LDL-cholesterol (mg/dl) (μ, SD)	100.4 (28.9)
HDL-cholesterol (mg/dl) (μ, SD)	39.4 (10.5)
C-reactive protein (mg/L) *	3.47 (1.23; 8.37)
Creatinine Clearance (mL/min) *	93.75 (78.20; 115.00)
Cystatin C (mg/L)*	1.03 (0.93; 1.19)
NT-proBNP (pg/mL)*	572.00 (279.90; 1101.00)
GRACE Score (points) (μ, SD)	95.9 (20.8)

* median, 25^th^ and 75^th^ quantile cut-point.

Table 2 shows the association of CYPC19*2 loss-of-function gene carrier status according to various sociodemographic, and clinical findings. Homozygous CYPC19C*2 carriers were more prevalent in males, most prevalent in the youngest category (8.7% in those 30–39 years), and more often belonged to the category with less than 10 years of school education. In addition current smokers had the highest prevalence of homozygous CYPC19*2 gene. However, the small numbers of homozygous carriers needs to be considered.

**Table 2:** CYP2C19C*2 allele status according to various sociodemographic characteristics, cardiovascular risk factors, and ECG findings at baseline

		**N**	CYP2C19C*2 allele status (row%) (AG or AA)	**p-value**	CYP2C19C*2 allele status (row%) (AA only)	p-value*
**All**		**1050**				
**Gender**	- Female	158	26.6		1.9	
	- Male	892	29.6	0.44	2.7	0.75
**Age (years)**	- 30-39	23	47.8		8.7	
	- 40-49	133	21.1		1.5	
	- 50-59	304	30.3		2.6	
	- 60-70	590	29.7	0.04	2.5	0.10
**School education**	- < 10 yrs.	625	31.2		3.8	
	- ≥ 10 yrs.	425	26.1	0.08	1.8	0.008
**Body mass index** (kg/m^2^)	- < 25	299	26.4		3.0	
	- 25-30	560	30.7		3.0	
	- >30	190	28.4	0.41	0.5	0.18
**Smoking status**	- Never	333	28.8		1.2	
	- Ex	664	29.2		3.2	
	- Current	53	30.2	0.98	3.8	0.34
**History of diabetes**	- Yes	184	28.8		2.7	
	- No	866	29.2	0.91	2.5	0.94
**History of MI**	- Yes	611	30.9		2.6	
	- No	439	26.7	0.13	2.5	0.31
**History of hypertension**	- Yes	583	29.5		2.7	
	- No	467	28.7	0.77	2.4	0.92
**History of heart failure**	- Yes	128	25.0		2.3	
	- No	891	29.6	0.28	2.6	0.58
**Angiographic score** (number of affected vessels)	- Zero/one	270	28.2		1.1	
	- Two	279	30.5		3.6	
	- Three	449	29.4	0.84	2.7	0.46
**Initial management of CHD**	- Conservative	193	25.4		1.6	
	- PCI	361	28.8		2.8	
	- CABG	496	30.9	0.36	2.8	0.68
Left ventricular function* (degree of impairment)	- No/only little	746	28.7		2.6	
	- Modest/severe	217	29.5		3.2	
	- Unknown	87	32.2	0.79	1.2	0.79
**Lipid-lowering agent**	- Yes	810	28.9		2.6	
	- No	238	29.8	0.78	2.5	0.96
**Beta-blocker**	- Yes	916	28.9		2.5	
	- No	132	30.3	0.75	3.0	0.87
**Calcium antagonist**	- Yes	86	27.9		3.5	
	- No	962	29.2	0.80	2.5	0.67
**ACE inhibitor**	- Yes	557	28.4		2.5	
	- No	491	29.9	0.58	2.7	0.86
**Diuretic**	- Yes	255	31.0		2.4	
	- No	793	28.5	0.45	2.7	0.68
**ASA**	- Yes	915	29.0		2.7	
	- No	133	30.1	0.79	1.5	0.69
**Clopidogrel or Ticlopidine**	- Yes	87	33.3		2.3	
	- No	963	28.8	0.37	2.6	0.59
**Proton Pump Iinhibitor (PPI)**	- Yes	66	33.3		0.0	
	- No	984	28.9	0.44	2.7	0. 25
**Clopidogrel or Ticlopidine or PPI**	- Yes	141	34.0		1.4	
	- No	909	28.4	0.17	2.8	0.18

*=Fishers Exact Test.

At entry into the inpatient rehabilitation n=257 (24.5%) patients received clopidogrel (prescribed in the acute care-hospital); at discharge from in-patients rehabilitation only n=80 (7.6%) received it. In addition, prescription went further down at 1 year follow-up (n=50, 4.7%) (data not shown).

Table 3 shows the age and gender adjusted mean values between CYP2C19C*2 loss of function gene carriers and laboratory measures. The only statistically significant differences were seen with total cholesterol and LDL cholesterol in the sense that patients with one CYP2C19*2 allele all had the highest values.

**Table 3:** Age and Gender Adjusted Mean Values of Body Mass Index and Various Biomarkers according to CYP2C19C*2 Allele Status

	CYP2C19C*2 allele status GG AG orAA		CYP2C19C*2 allele status GG AG AA	
		**p-value**		**p-value**
Body mass index (kg/m^2^)	27.5		27.5	0.39
	27.6	0.62	27.7	
			26.9	
Total cholesterol [mmol/L]	173.3		173.3	0.04
	178.5	0.02	179.0	
			172.9	
HDL cholesterol [mmol/L]	40.5		40.5	0.29
	41.6	0.12	41.7	
			41.3	
LDL cholesterol [mmol/L]	101.2		101.2	0.10
	105.1	0.05	105.6	
			100.4	
Leukocytes [Giga/L]	7.1		7.1	0.37
	7.3	0.31	7.4	
			6.8	
C-reactive protein* [mg/L]	2.74		2.74	0.60
	2.71	0.88	2.77	
			2.12	
Interleukin-6 * [pg/mL]	3.12		3.12	0.62
	3.01	0.47	3.04	
			2.76	
Creatinine clearance [ml/min]	110.2		110.2	0.61
	110.9	0.69	111.3	
			106.5	
Cystatin C * [mg/L]	1.03		1.03	0.53
	1.01	0.27	1.01	
			1.00	
NT-proBNP* [pg/mL]	414.9		414.9	0.22
	468.8	0.08	468.4	
			473.6	

*geometric means.

**Figure 1:**
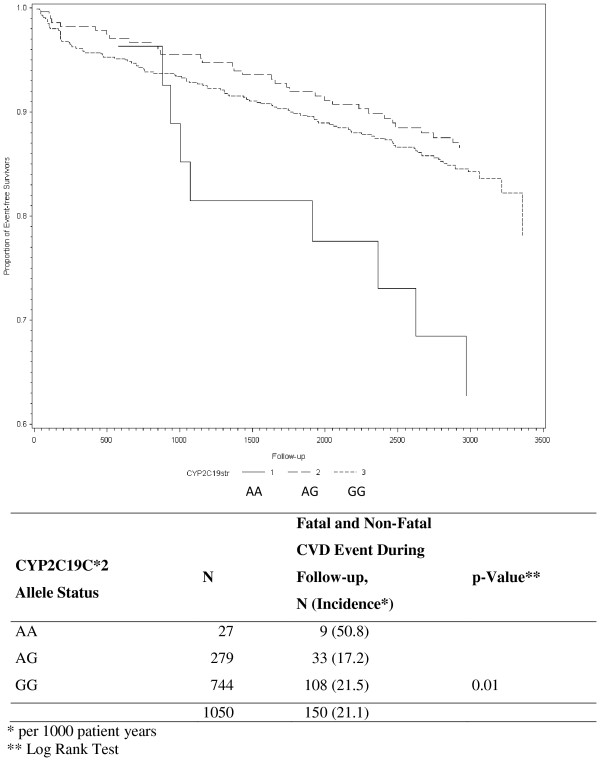
Kaplan-Meier Estimates of Secondary Fatal and Non-Fatal CVD Events During Follow-Up (time=days) According to CYP2C19C*2 Allele Status.

**Figure 2:**
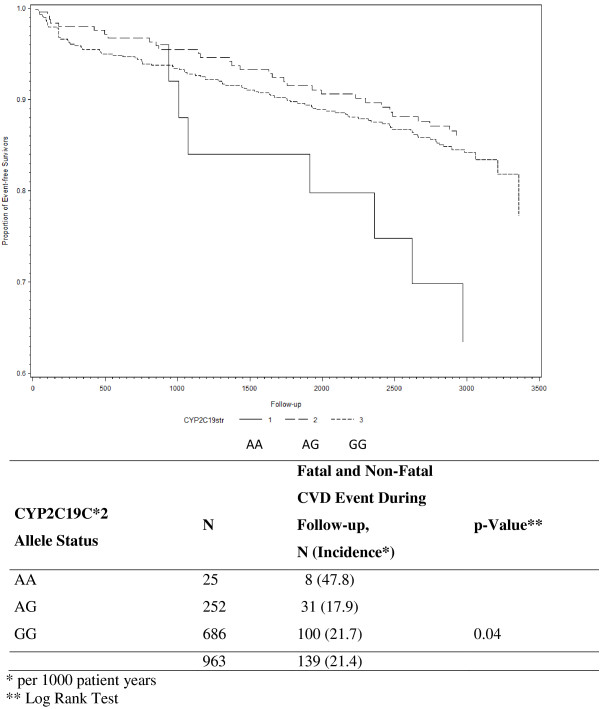
Kaplan-Meier Estimates of Secondary Fatal and Non-Fatal CVD Events During Follow-Up (time=days) According to CYP2C19C*2 Allele Status in Patients with no Intake of Clopidogrel or Ticopidine at Baseline and One Year Follow-up).

During a mean follow-up of 8.1 years, 150 patients (14.3%) experienced a secondary CVD event (n=59 (39%) CV death, n=50 (33%) nonfatal MI, and n= 41 (27%) non-fatal stroke). Figure 1 shows Kaplan-Meier survival curves of time to secondary CHD event in patients with and without CYPC19*2 loss-of-function gene. Overall, patients homozygous for the CYP2C19*2 loss-of-function gene had the highest incidence rate (50.8 per 1000 patient years) (log rank test: p=0.01). After exclusion of all patients with a clopidogrel and ticlopidine intake at baseline and one-year follow-up (n=87, 80 received clopidogrel and 7 received ticlopidine) the results were quite similar (p=0.04) (Figure 2).

Table 4 shows the results of multivariable analysis. Homozygous carriers of the CYP2C19C*2 allele showed a HR of 2.59 (95% CI 1.27-5.28) after adjustment for covariates, whereas patients with one CYP2C19*2 allele showed no increased risk (the additional adjustment for CRP and Cystatin C increased the risk for homozygous carriers of the CYPC19C*2 to 2.76 (95% CI 1.35-5.66), but no meaningful change was seen for the heterozygous ones). If treatment with clopidogrel or ticlopidine was considered in homozygous carriers of the CYP2C19C*2 allele only 2 patients were affected of whom one showed a cardiovascular event during follow-up.

**Table 4:** Association of CYP2C19C*2 allele status with fatal and non-fatal cardiovascular events during follow-up

	**Results of multivariable analysis**	
	**HR (95% CI) Adjusted for age and gender**	**HR (95% CI)** *Adjusted for multiple covariates
CYP2C19C*2 Allele status		
GG	1 referent	1 referent
AG	0.79 (0.54-1.17)	0.80 (0.52-1.23)
AA	2.37 (1.20-4.68)	2.59 (1.27-5.28)
	p for trend 0.66	p for trend 0.45
**After exclusion of patients on clopidogrel or ticlopidin (n total = 963):**		
CYP2C19C*2 Allele status		
GG	1 referent	1 referent
AG	0.82 (0.55-1.23)	0.87 (0.56-1.35)
AA	2.18 (1.06-4.49)	2.27 (1.06-4.86)
	p for trend 0.67	p for trend 0.42

* adjusted for age, gender, BMI, school education, rehabilitation clinic, smoking status, history of diabetes mellitus, PCI, CABG, left ventricular function**,** HDL-cholesterol, LDL-cholesterol, treatment with lipid-lowering drugs, NT-proBNP.

Adjustment for co-administration of PPI and calcium antagonists, which also act as substrate and inhibitor for CYP2C19, only marginally changed the prognostic value of the CYP2C19 *2 allele and showed no association with the outcome. Also if PPI and calcium antagonists were considered as simultaneous medication, no increase of risk was seen, but it has to be considered that only 4 patients had this combination (data not shown). Patients who did undergo stenting during initial treatment showed similar risk patterns (HR for homozygous carriers of the CYPC19C*2 was 2.78 (95% CI 1.01-7.69) as the overall study population. After exclusion of patients on clopidogrel or ticlopidin homozygous carriers of the CYP2C19C*2 allele showed still a HR of 2.27 (95% CI 1.06-4.86) after adjustment for covariates.

Table 5 shows that the addition of CYP2C19 information to the risk prediction model did not add incremental information to parameters of model fit, discrimination and calibration, not demonstrating any clinical utility in this patient population.

**Table 5:** Measures of model accuracy with and without CYP2C19C*2 status information (n=1050)

	Basic model*	**Basic model plus CYP2C19 polymorphism**
**Model fit**		
LR	69.25 (df=15, p<0.0001)	69.80 (df=16, p<0.0001)
AIC	1603.8	1605.2
BIC	1646.1	1650.3
**Discrimination**		
C-statistic (AUC, 95% CI)	0.693 (0.645-0.742)	0.695 (0.647-0.744)
**Calibration**		
Integrated discrimination improvement (IDI)		0.00016 (p=0.87)
Net reclassification improvement (NRI)		−1.3% (p=0.60)

* adjusted for age, gender, BMI, school education, , rehabilitation clinic, smoking status, history of diabetes mellitus, PCI, CABG, left ventricular function**,** HDL-cholesterol, LDL-cholesterol, treatment with lipid-lowering drugs, NT-proBNP [pg/mL].

## Discussion

In this medium-size cohort study of patients with stable coronary heart disease at baseline only few patients homozygous for a loss-of-function variant of the CYP2C19*2, allele, compared to the wild-type carriers, showed a statistically significant higher risk for secondary CVD events during long-term follow-up even after adjustment for covariates. The increased risk was independent of the co-administration of PPIs, which may lead to further functional variability in the P450 isoenzyme activity. An increased risk was also suggested by the CYP2C19*2 genotype alone, respectively in patients without clopidogrel (or ticlopidine) intake. Results of this study suggest that, although the CYP2C19*2 genotype alone may eventually carry independent prognostic information and may be useful in identifying patients at increased risk for subsequent complications, the fact that very few patients were affected with the homozygous loss-of-function has to be considered. The latter makes it difficult to demonstrate a clinical benefit of genotyping in this patient population.

Our data add to the evidence from clinical trial populations indicating an increased risk in carriers of the CYP2C19*2 loss-of-function allele compared to non-carriers taking clopidogrel [3,6,11]. Although the prevalence of the homozygous carriers was low in our populations and the clinical relevance for the overall population of patients with stable CHD may be limited, the associated risk with a secondary CVD event for the few affected patients may be high. However, today, all patients after drug eluting stent implantation may be on P2Y12 inhibitors, predominantly on clopidogrel. We did not find an increased risk for the heterozygotes with one normal CYP2C19*2 allele. This is in line with the results of a meta-analysis of Holmes and colleagues [12] who compared individuals with any copy of CYP2C19 genetic variants (alleles *2 through *8); presumably, the vast majority of subjects carried a heterozygous genotype. Although they found a higher risk for CVD events of 1.18 (95% CI 1.09-1.28) using fixed-effects models and of 1.34 (95% CI 1.15-1.56) for a random effects model, there was a trend towards the null in larger studies consistent with small study bias and they concluded that there was no association of genotype with CVD events. Unfortunately, they did not further characterize the risk of the homozygous carriers. A very recent study conducted in n=506 patients with CAD from China also found an additional increased risk for adverse cardiovascular outcomes independent from taking clopidogrel, which was still present but was considerably reduced in the second year of follow-up [13]. Various alleles have been identified for CYP2C19 of which several combinations result in different metabolizer phenotypes [14]. The most common CYP2C19*2, which was the subject of our study, is present in 95% of the patients with a poor metabolizer phenotype [3]. We did not observe an adverse association of the loss-of-function gene carrier status with other concurrent CVD risk factors such as diabetes, lipid values, inflammatory parameters, or markers of renal function, making the probability of confounding by these risk factors unlikely. In addition all of them had been controlled for in the final multivariable analysis. Unfortunately we have no measure of platelet activity included to further demonstrate the pharmacogenetic and pharmacokinetic effects of CYP2C19*2. In patients of Caucasian origin undergoing PCI (n=738) carriers of the *2 allele showed a significantly higher adenosine diphosphate (ADP)-induced platelet aggregation and the prevalence of this allele was 1.9% [15], which is a little lower than in our study. The study of Shuldiner and colleagues in 429 Amish generally healthy white participants found that the CYP2C19*2 genotype was associated with a diminished platelet response of clopidogrel on inhibition of ADP induced platelet aggregation [16]. In a separate population of 227 patients undergoing PCI between 2004 and 2007 the study showed that carriers of the CYP2C19*2 allele had twice the risk of having a cardiovascular event during the one-year follow-up.

Our study results are in line with the recent Black Box Warning issued by the Food and Drug Administration (FDA) that patients carrying the CYP2C19*2 allele should receive other antiplatelet medication than clopidogrel or alternative dosing strategies [8]. It has been suggested that other anti-platelet medication such as prasugrel or ticagrelor in combination with the CYP2C19*2 allele may not result in an impaired prognosis [11]. Future studies, however, have to investigate whether the increased risk of loss-of-function allele carriers can be decreased in general by using other pharmacologic strategies in routine clinical care. As the CYP2C19*2 genotype may only account for about up to 12% of the clopidogrel response [16], the higher percentages may only be found in homogeneous populations, other relevant factors such as diabetes or age may play a role, too. Although the bedside-genotyping may be of additional value to gather prognostic information, especially for the few homozygous CYP2C19*2 loss-of-function carriers, as the association with an adverse cardiac outcome seems evident for these patients, the consequences for a modified treatment schedule are not clear yet.

We did not find an interaction of the CYP2C19 polymorphism with concomitant intake of PPIs, known to be strong inhibitors of the CYP2C19 activity and therefore may increase the risk of thrombosis [17]. Although a pharmacokinetic and pharmacogenetic interaction has been described, the clinical consequences of this combination and the relationship to adverse clinical outcomes such as secondary CVD-events seem to be minimal or non-existent [18]. This assessment is supported by our observations, although the limited sample size and the relatively rare use of clopidogrel a decade ago in this routine clinical care setting of our study population may only allow excluding a strong effect. We did also not find an association with the outcome of PPIs and simultaneous administration of calcium antagonists, a finding very recently described in a study including patients with elective PCI and one year follow-up [19].

### Limitations

We did not include other genetic polymorphisms that are related to metabolic changes in platelet activation. However, the carriage of *2 allele has been described in 95% of poor metabolizers [3]. Furthermore, this study population consisted mostly of Caucasian, male patients (85.0%), aged 50 – 70 years at baseline. Therefore, results may have limited generalizability to females, younger adults, or especially other ethnic groups as we know that there is large variability for SNPs between ethnic groups. However, the internal validity of our findings should not be affected. In addition, we considered clopidogrel use at baseline and one year follow-up, but the low prevalence probably reflects clinical practice a decade ago.

## Conclusions

Despite these limitations we conclude that in patients with stable CHD homozygous carriers of the loss-of-function allele CYP2C19*2 may be at increased risk for subsequent CVD events, which may be especially high in patients on clopidogrel. Whether early identification of the few affected patients may allow a treatment strategy with improved outcomes should be investigated further. However, the fact that few patients carrying the homozygous loss-of-function variant has to be considered if assessing the clinical utility and benefit of genotyping in this patient population.

## Abbreviations

ACE: Angiotensin-converting-enzyme; ACS: Acute coronary syndrome; ASA: Acetylsalicylic acid; CHD: Coronary heart disease; CABG: Coronary artery bypass grafting; CYP2C19: Cytochrome P450, family 2, subfamily C, polypeptide 19); CVD: Cardiovascular disease; HR: Hazard ratio; PCI: Percutaneous coronary intervention; PPI: Proton pump inhibitor.

## Competing interest

Iris Rajman is a full-time employee of Novartis Institute for Biomedical Research. Dietrich Rothenbacher has been a full-time employee of Novartis Pharma until November 2010.

## Authors’ contribution

DR and IR had conceived the study and drafted the manuscript. MMH organized and coordinated the genotyping. WK organized and coordinated the other laboratory measurements. DR performed the statistical analysis. LPB, WK, and HB participated in the design and conduct, and coordination of the study. All authors read and approved the final manuscript.

## Pre-publication history

The pre-publication history for this paper can be accessed here:

http://www.biomedcentral.com/1471-2261/13/61/prepub
